# Preparation and characterization of decellularized bovine bone as a bioscaffold for bone tissue engineering applications

**DOI:** 10.22038/ijbms.2025.87816.18969

**Published:** 2026

**Authors:** Roya Akbarpour, Majid Salehi, Simin Nazarnezhad, Ghasem Abbaszadeh-Goudarzi

**Affiliations:** 1 Student Research Committee, School of Medicine, Shahroud University of Medical Sciences, Shahroud, Iran; 2 Regenerative Medicine Research Center, Shahroud University of Medical Sciences, Shahroud, Iran; 3 Department of Tissue Engineering, School of Medicine, Shahroud University of Medical Sciences, Shahroud, Iran; 4 Metabolic Syndrome Research Center, Mashhad University of Medical Sciences, Mashhad, Iran; 5 Tissue Engineering Research Group (TERG), Department of Anatomy and Cell Biology, School of Medicine, Mashhad University of Medical Sciences, Mashhad, Iran; 6 Department of Medical Biotechnology, School of Medicine, Shahroud University of Medical Sciences, Shahroud, Iran

**Keywords:** Alendronate, Bone tissue engineering, Crocin, Decellularized bone – scaffolds, Osteogenesis

## Abstract

**Objective(s):**

This study aimed to develop and evaluate decellularized bovine bone (DBB) scaffolds and investigate their potential to promote osteogenic differentiation when combined with Crocin and Alendronate.

**Materials and Methods:**

Bovine bone was decellularized using a combination of physical (freeze–thaw cycles, sonication), chemical (sodium dodecyl sulfate), and enzymatic (deoxyribonuclease I) treatments to preserve native bone architecture. Scaffold properties were assessed by evaluating extracellular matrix (ECM) integrity and compressive strength. Biocompatibility was confirmed through cytotoxicity and hemolysis assays. *In vitro* osteogenesis was analyzed using alizarin red staining and qRT-PCR (quantitative real-time polymerase chain reaction) to quantify expression of osteogenic markers RUNX2, osteocalcin, osteopontin, and osteonectin following treatment with crocin (Cr 5 mg/ml), Alendronate (ALN 1 mg/ml), and their combination (Cr/ALN 5 mg/ml).

**Results:**

DBB scaffolds-maintained ECM structure and compressive strength (14.56 ± 0.82 MPa), comparable to native bovine bone (17.86 ± 0.14 MPa). No cytotoxic or hemolytic effects were observed. Crocin, Alendronate, and Cr/ALN treatments significantly enhanced RUNX2 expression (70%, 60%, and 65%, respectively), while Osteocalcin expression increased in Cr (50%) and Cr/ALN (25%) groups. Osteopontin and osteonectin expression also rose in Cr and Cr/ALN groups, supporting enhanced osteogenic differentiation.

**Conclusion:**

Based on *in vitro* findings, DBB scaffolds demonstrate favorable mechanical and biological properties, and loading the scaffolds with crocin and Alendronate enhanced osteogenic differentiation and matrix mineralization, indicating potential for bone-regeneration applications.

## Introduction

It is well-known that bone defects exceeding the critical size require stimulation of bone repair and regeneration. In this sense, bone substitutes have emerged as promising candidates to regenerate the lost bone tissue (1). Although autograft is still recognized as the Gold Standard, it encounters various challenges, such as lack of available bone at the donor site, increased risk of infection, and the necessity for additional surgeries (2). Allografts are limited in use due to the risk of viral infection transmission and immunological rejection (3). Xenograft fulfills most of the requirements of a graft, such as being osteoinductive, osteoconductive, having an unlimited supply, being mechanically strong, and being biodegradable. Thus, progress in biomaterial research for bone regeneration promotes the utilization of xenogeneic bones (4, 5).

Using bovine cancellous bone as a xenotransplant has the potential long-term risk associated with the transfer of xenogenetic material to the recipient; however, the development of a reliable strategy to prevent host immunological and inflammatory reactions to the cancellous bone graft could provide an available, plentiful, and cost-effective source of materials (6). Among the different techniques available, decellularization stands out as the most effective technique to achieve this goal (7).

Decellularization is a procedure that removes all cells and genetic components from tissue while preserving components of the extracellular matrix (ECM) like collagen, glycosaminoglycan, glycoproteins, bioactive molecules, cytokines, and growth factors (8). Hence, the biological and physicochemical characteristics of decellularized bone tissue are preserved, offering structural support and biological signals to facilitate cell attachment, growth, and differentiation within the scaffold (9). Decellularized bone has features such as osteoconduction, osteoinduction, and osteointegration; hence, it is introduced as a scaffold that mimics the natural bone structure (10).

According to the literature, there are two essential criteria for decellularization: (I) the residual genetic material should not exceed 50 ng/mg of the dry weight of the tissue, (II) the ECM should preserve its integrity (11).

Decellularization techniques encompass a range of physical, chemical, and enzymatic processes that utilize detergents, enzymes, and temperature to disrupt and eliminate cells. These approaches successfully minimize immune reactions in the host tissue (12). The presence of fat in bone grafts can also lead to the risk of bone resorption and fibrosis due to the reaction of giant cells. To minimize this risk, it is crucial to perform delipidation at the beginning of the decellularization process (13). During decellularization, detergents like sodium dodecyl sulfate (SDS) are frequently employed. This detergent effectively removes cells and denatures the protein structures (14). 

Alendronate (ALN) is the most common form of bisphosphonate that is mainly utilized in the treatment of osteoporosis and bone abnormalities. It effectively inhibits bone resorption through the inhibition of osteoclast activity, while concurrently enhancing ossification by promoting the proliferation and maturation of osteoblasts. Additionally, ALN enhances bone mineral density (15, 16).

Crocin, a natural bioactive pivotal component of saffron, exhibits a range of pharmacological effects, including an inflammation reducer, cancer treatment, free radical damage, hypolipidemia, and anti-depression. Also, crocin demonstrates osteoinductive and osteoconductive properties, and it is effective in improving rheumatoid arthritis and osteoporosis. Recent research indicates that crocin can promote osteoblast differentiation of BMSCs (17-20). 

So far, many different approaches have been introduced for bone decellularization techniques. However, a consensus on the optimal decellularization methods has yet to be reached. (21). In this study, we developed a dual-functionalized decellularized bovine bone (DBB) scaffold using a novel multi-step protocol that combines physical, chemical, and enzymatic treatments to successfully remove cellular and lipid residues while maintaining the ECM and mechanical strength. Following decellularization, the scaffold was loaded with crocin, an osteoinductive and anti-oxidant carotenoid, and ALN, an anti-resorptive bisphosphonate, to achieve a synergistic effect by stimulating osteoblast differentiation and suppressing osteoclast-mediated bone resorption. Comprehensive *in vitro* assessments of biocompatibility, drug release behavior, and morphological and histological features demonstrated a controlled, sustained release of both bioactive agents without an initial burst, overcoming limitations of previous scaffolds that lacked multifunctionality or relied on single-drug loading. This multifunctional design offers a structurally stable and biologically active scaffold with promising potential for bone tissue engineering applications.

## Materials and Methods

### Processing of bovine bone

Fresh bovine femoral heads, sourced from surplus materials provided by a licensed local slaughterhouse, were used in this study, and no live animals were involved. The soft tissue was carefully separated from the bone tissue. The femur bones were rinsed in running water for 1 hour and subsequently sectioned into small fragments (about 0.5 × 0.5× 0.5 cm) These bone fragments were then immersed in a deionized water solution containing 5,000 units/ml of heparin (Sigma-Aldrich, USA), 1% Penicillin/Streptomycin (P/S) (Gibco, US), and 1% Gentamicin (Gibco, US) for a duration of 24 hr. Following this, the fragments were rinsed with 800 ml of a 0.9% saline solution and preserved at −80 °C until required (22).

### Decellularization of cancellous bone

The decellularization of bovine bone fragments was conducted using previous studies with modifications (23). five freeze-thaw cycles (each cycle consists of 1 min in liquid nitrogen (-196 °C) and 5 min in hot water at 56 °C). After freeze-thaw cycles, bone fragments were ultrasonicated individually for two hours at 20 kHz. Then, the samples were washed in SDS (Merck Millipore, Germany) at different concentrations: 1% for 24 hr, 0.1% for six hours, and 0.01% for six hours on an orbital shaker at 150 rpm. Bone samples were washed in DW for 24 hr, then the lipids were extracted with a 1:1 mixture of chloroform and 100% ethanol (absolute ethanol) (Sigma-Aldrich, USA) for 24 hr at RT, under shaking at 150 rpm. To remove remaining chloroform and ethanol, bone samples were washed in DW for 24 hr. The samples were treated with DNase I (15 IU/ml) (yektatajhiz, Iran) for 24 hr at 37 °C with continuous shaking utilizing a magnetic shaker. After decanting the enzymatic solution, bone fragments were ultrasonicated again and washed in hydrogen peroxide 3% (H_2_O_2_) (Merck Millipore, Germany) for two hours. Finally, decellularized bovine bone fragments underwent treatment with 0.1% peracetic acid 0.1% (Merck Millipore, Germany) for four hours.

### Morphologies

The pore structure and surface elemental analysis using scanning electron microscopy and energy-dispersive X-ray spectroscopy (SEM-EDS) were characterized. By using an ethanol solution, the dehydration of scaffolds was accomplished. Scaffolds were sputter-coated lightly with gold, and Imaging was performed using a Hitachi scanning electron microscope (Hitachi, Ltd., Tokyo, Japan).

### Measurement of DNA concentration

DNA quantification was performed by isolating DNA from DBB and native bovine bone (NBB) tissue following the manufacturer’s guidelines, utilizing a commercially available extraction kit QIAamp DNA Blood & Tissue Kit (Qiagen, Germany). The total DNA was measured using a spectrophotometer (NanoDrop 1000; Thermo Fisher Scientific, Inc.) at 260 nm (24).

### Biomechanical testing

Compressive strength was used to estimate the biomechanical resistance of NBB and DBB.The fragments were sectioned into rectangular shapes with 10 mm length and 5 mm width, and compression testing was assessed using a universal device (SANTAM STM, Iran). A 5 kN load cell was employed for the analysis. The speed of the crosshead was 3 mm/min, and loading pressure was applied to the samples until they cracked. 

The fatigue test is used to evaluate the durability of NBB and DBB under repeated loading conditions. Force was applied at a speed of 0.01 mm/min (ASTM E1942), 5 Hz at room temperature, and this force continued until the appearance of the first crack in the samples.

### Examination of weight loss

To determine the weight loss, firstly, NBB and DBB were recorded. Then, the scaffolds were immersed in PBS (Phosphate-buffered saline) (Kalazist, Iran) and maintained at 37 °C for 63 days. At set time points (1, 2, 3, 7, 14, 21, 28, 35, 42, 49, 56, and 63 days), the samples were extracted from the PBS solution, allowed to dehydrate, and their final weight was measured. The percentage of weight loss was computed based on the following equation, in which the initial dry weight (W0) and final dry weight (W1) of each sample were used.

Weight loss (%) = [W_0_ – W_1_/W_0_] × 100

### Porosity test

The porosity of the scaffolds was assessed using the immersion of scaffolds in alcohol. Approximately 3 ml of alcohol was poured into a graduated cylinder, and the initial volume was carefully measured. The scaffolds were submerged in the alcohol, and their secondary volume was noted. After a 30-second interval, the scaffolds were carefully removed from the alcohol, and the ultimate volume was documented. The porosity percentage was computed by the equation below:

$v_{1}-v_{2}/v_{2}-v_{1}\times 100\%$

### Histological analysis

Samples of NBBs and decellularized bones were fixed in 10% formalin solution (Merck Millipore, Germany) for 48 hr, rinsed with distilled water, and decalcified with 3% and 5% nitric acid (Temadkala, Iran), and it was changed after 24 hr. The completion of decalcification was assessed by the flexibility and pin penetrability of the bone, then paraffin-embedded and cut into 5 μm thick sections using a cryotome (Thermo Scientific). The sections were stained with hematoxylin and eosin (H&E) (Merck Millipore, Germany), Masson’s trichrome (Sigma-Aldrich, USA), and 4’,6-diamidino-2- phenylindole (DAPI) (Sigma-Aldrich, USA). Images of sliced samples were obtained using a microscope.

### Alendronate and crocin release assay

ALN (Sigma-Aldrich, USA) and crocin (Sigma-Aldrich, USA) were loaded in DBB scaffolds at concentrations of 1, 3, and 5 mg and incubated in 4 ml of PBS at 37 °C for 1, 2, 4, 6, 12, 24, 48, 72, 120, 144, 168, 336, 504, and 672 hr. At each time point, 200 µl of solution was extracted for analysis, and the same amount of fresh solution was added to the main solution. Optical density was measured at 260 nm for crocin and 280 nm for ALN using a microplate reader (Thermo Fisher Scientific), and the data were compared to the ALN and crocin standard curves in PBS (25, 26).

### Cytotoxicity assay

The MTT assay was used to assess the cytotoxicity of the DBB scaffold. This procedure was done using an indirect test under certain conditions, employing the MC3T3-E1 cell line, a mouse calvaria-derived pre-osteoblastic model (27)**.** Briefly, MC3T3-E1 (3×) were seeded in 96-well cell culture plates in Dulbecco’s Modified Eagle’s Medium (DMEM, Gibco, US) supplemented with 10% (v/v) fetal bovine serum (FBS, Gibco, US) and 1% Penicillin/Streptomycin. They were incubated under controlled conditions (5% CO2, 37 °C) for 24 hr. DBB scaffolds fragment measuring approximately 0.5 × 0.5 × 0.5 cm (corresponding to ~50 mg dry weight) were incubated with ALN, crocin, and the combination of both ALN and crocin at concentrations of 1, 3, and 5 mg (28, 29). After 24 hr, the scaffolds were removed, and 100 µl of conditioned media was added to the seeded MC3T3-E1, and the cytotoxicity was assessed using the MTT assay at 48 and 72 hr. Ten microliters of 5 mg/ml MTT was added to each well and incubated for four hours at 37 °C. The supernatant was discarded, and 100 μl of dimethyl sulfoxide (DMSO) was added to the wells. After 20 min, the samples were evaluated with a plate reader at a wavelength of 570 nm.

The calculation of cytotoxicity was performed using the following formula:

Viability (%) = (OD _sample_ / OD _control_) × 100 

### Blood compatibility evaluation

At first, NBB and DBB were placed in microtubes. 2 ml of fresh anti-coagulated human blood was diluted with 2.5 ml of 0.9% normal saline. Then, 200 µl of diluted blood was added to each microtube. After incubation for 60 min at 37 °C, the samples were centrifuged for 10 minutes at a speed of 1500 rpm. The supernatant was conveyed to a 96-well plate, and the absorbance of the samples was assessed at 545 nm. Blood diluted in deionized water and normal saline was considered the positive and negative control, respectively. The subsequent equation was employed to determine the percentage of hemolysis (HD). 

HD (%)=[(Ds _sample_ –Dn _negative control_)/ (Dp _positive control_ - Dn)] ×100

### Osteogenesis assay

Alizarin red staining (ARS) quantification of calcium phosphate (hydroxyapatite) deposition in MC3T3-E1 osteoblastic cell cultures, providing evidence of their differentiation into mature bone-forming cells. MC3T3-E1 cells were cultured in 6-well plates and treated with conditioned media of drug-loaded DBB scaffolds, which contain ALN, crocin, and the combination of ALN/crocin, DBB, and the control group over 7 days. Throughout the experimental duration, the conditioned medium was refreshed every 3 days. The treated cells were fixed in 10% Formaldehyde (Merck Millipore, Germany) for 15 min at room temperature, carefully removed the fixative and rinsed the cells three times with distilled water, and stained with 1 ml/well Alizarin Red Stain Solution. Incubated at room temperature for 20 min (30). 

### Gene expression analysis using RT-qPCR

MC3T3-E1 cells were cultured for 7 days and assessed for the expression of osteogenic genes, including Runt-related transcription factor 2 (RUNX-2), osteopontin (OPN), osteocalcin (OC), osteonectin (OSN), and β-actin as control. RNA was isolated using an RNA extraction kit (Denazist, Iran) following the manufacturer’s instructions, and the concentration of the RNA samples was determined with a NanoDrop. cDNA was generated utilizing a cDNA synthesis kit (Denazist, Iran). The sequences of the primers are detailed in Table 1. The quantitative PCR (qPCR) amplification commenced with an initial denaturation step at 95 °C for 10 min, followed by 40 cycles of 95 °C for 30 sec, 60 °C for 1 min, and 72 °C for 1 min. The reactions were conducted using the real-time PCR system (Roche LightCycler 96, Germany), and the results were analyzed using the ΔΔCt relative gene expression normalized to β-actin (31).

### Statistical analysis

The outcomes derived from the examined groups at each phase were evaluated utilizing the one-way ANOVA statistical test using GraphPad Prism version 8 software. Each experiment was conducted with at least three repetitions. The data are presented as mean ± standard deviation (SD), and the results were considered statistically significant with a *P*-value of 0.05.

## Results

### Evaluation of SEM-EDS

**Figure 1:**
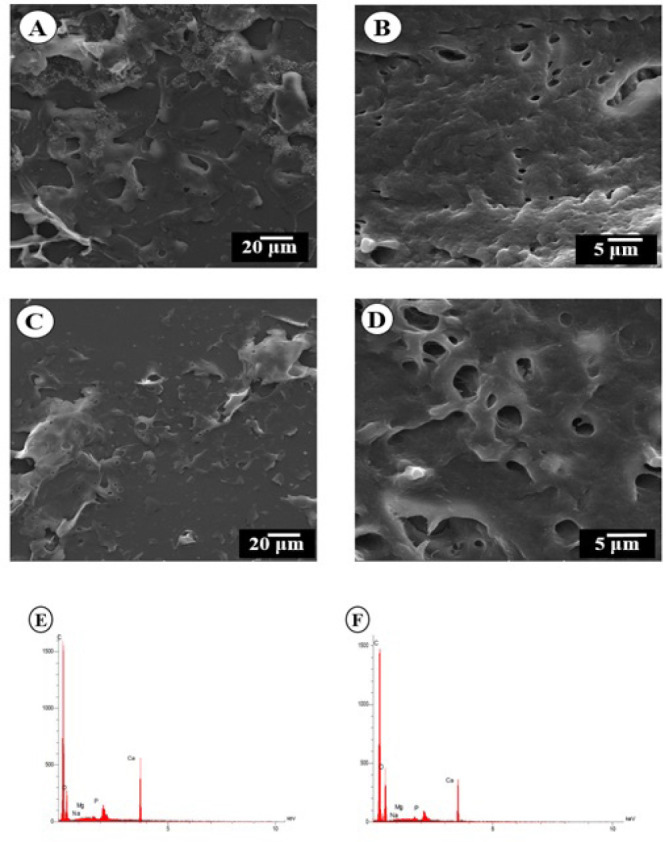
Evaluation of Scanning electron microscopy (SEM) and energy-dispersive X-ray spectroscopy (EDS) analysis of native and decellularized bovine bone (NBB and DBB)

SEM demonstrates noticeable differences in the surface structures of NBB and DBB (Figure 1). DBB maintains its typical structure, with the presence of collagen fibers and minerals, while lipid components and bone cells have been eliminated. In contrast, NBB shows a compact surface morphology and the presence of fat cells. Additionally, DBB has more porosity compared to NBB. The findings suggest that this protocol effectively removes fat and cells while preserving the microscopic structure of the ECM, similar to native bone. This is crucial for maintaining the bone’s ability to promote differentiation and bone growth.

EDS surface analysis of element composition revealed the presence of carbon, sodium, calcium, phosphorus, and oxygen in the NBB and DBB scaffolds (Figure 1 E, F). A decrease in the amount of calcium in NBB compared to DBB scaffolds. The amount of carbon, phosphorus, magnesium, and sodium in DBB scaffolds was closer to NBB.

### DNA quantification and DAPI staining

**Figure 2:**
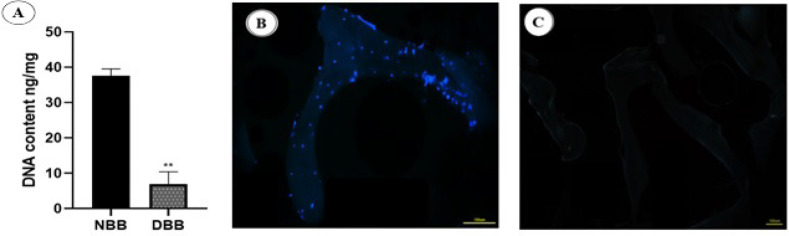
DNA quantification and DAPI staining confirming effective decellularization

To ensure the accuracy of decellularization, the content of DNA in NBB and DBB was extracted and compared. As illustrated in Figure 2A, the mean total DNA content in NBB scaffolds was 39.5 ± 1.5 ng/mg dry weight of ECM; this value significantly decreased to 10.2 ± 2.3 ng/mg dry weight of ECM in DBB scaffolds. 4,6- 4,6-diamidino-2-phenylindole (DAPI) staining displayed that Cell nuclei are absent in the DBB scaffold, while many cell nuclei are marked with blue dots in NBB scaffolds (Figure 2B, C). The results of DNA extraction and DAPI staining show that the decellularization process was done well. 

### Histological analysis

**Figure 3:**
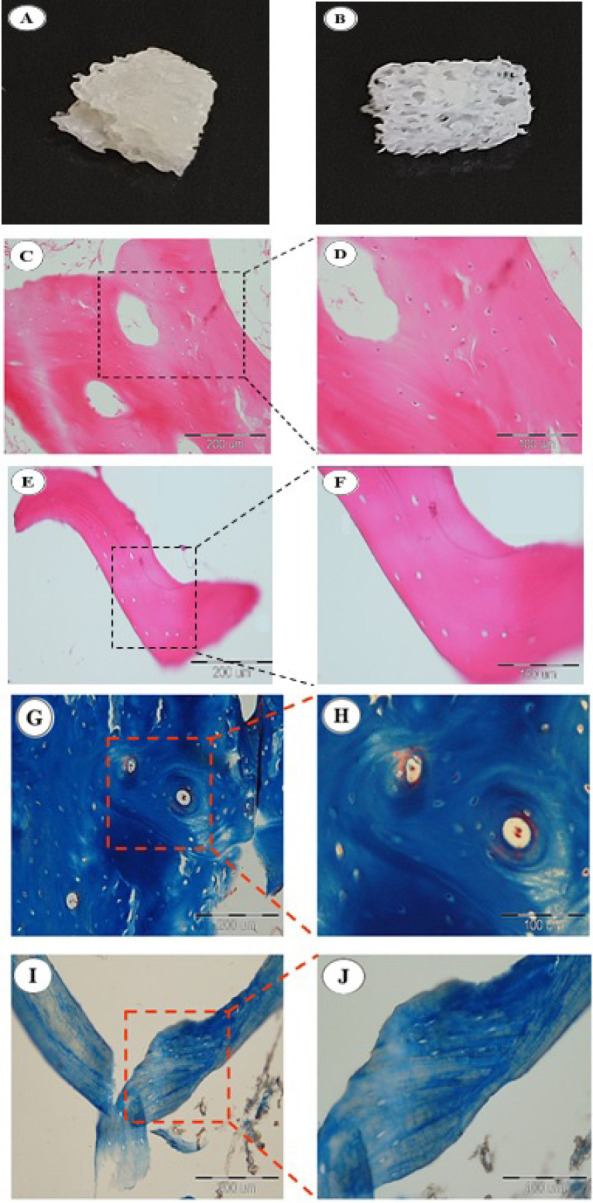
Histological analysis of native and decellularized bovine bone scaffolds (NBB, DBB)

After the decellularization process, red bone marrow and fat were eliminated from spaces between the cancellous bone structure, revealing the porous morphology (Figure 3A and B). Additionally, histological analysis demonstrates that DBB scaffolds are remarkably absent of cellular bone matrix and adipocytes compared to the NBB scaffolds. Also, the naturally porous structure of the extracellular bone matrix was successfully preserved in the DBB scaffolds (Figure 3C- F).

Masson’s Trichrome staining indicated that the cytoplasm was entirely removed, as no red areas were present, while the collagen, which stained blue, was preserved effectively (Figure 3 G-J).

### Biomechanical analysis

#### Compressive strength

**Figure 4:**
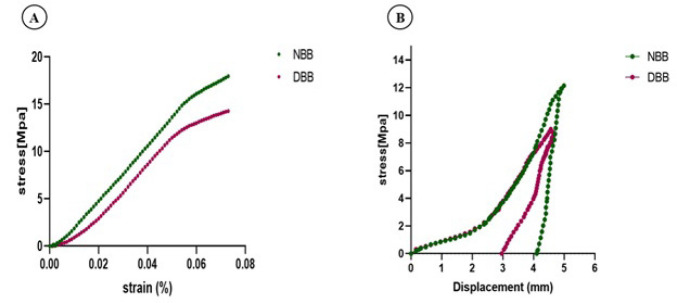
Biomechanical analysis of native and decellularized bovine bone scaffolds (NBB, DBB)

Following decellularization, there was no dramatic change in mechanical strength. Before decellularization, the compressive strength of NBB was 17.86 ± 0.14 MPa, while after decellularization, the compressive strength of DBB was 14.56 ± 0.82 MPa (Figure 4A). The compressive strength of the DBB scaffold was close to that of NBB. This indicates that the decellularization process did not significantly affect the mechanical properties of the bone matrix, and the mechanical strength of DBB was perfectly maintained.

### Fatigue test

The test was conducted cyclically; thus, the indenter tip was pressed into the same location repeatedly to keep the scaffolds in the testing position. A constant minimal load was maintained between cycles. The maximum load applied was 12.15 ± 0.35 MPa for NBB and 9 ± 1.06 MPa for DBB; the first complete cycle of indentation testing for both NBB and DBB is illustrated in Figure 4B.

### Assessment of porosity

**Figure 5:**
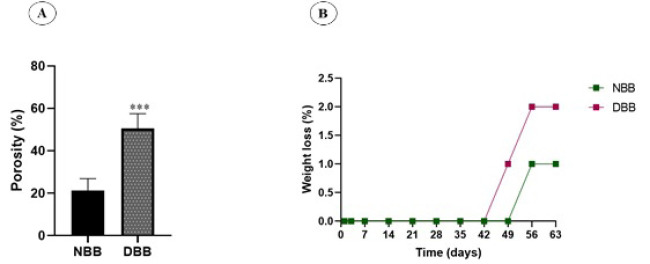
Porosity and weight loss assessment in native and decellularized bovine bone scaffolds (NBB , DBB)

The porosity of the scaffolds was assessed through a liquid displacement technique. Comparison between NBB and DBB scaffolds revealed that the decellularization process led to an increase in the porosity of the scaffolds (29.31 ± 4.02; *P*<0.001; Figure 5A). High porosity is sufficient for cell penetration and migration.

### Weight loss analysis

Degradability was assessed via the long-term weight loss assay in PBS over 63 days. The weight loss of scaffolds at various time intervals (1, 2, 3, 7, 14, 21, 28, 35, 42, 49, 56, and 63 days) is shown in Figure 5B. After soaking in PBS for 63 days, the weight loss percentages of NBB and DBB were measured. 1± 0.12 and 2 ± 0.5 %, respectively (n = 5; *P*<0.0001).

### Drug release

**Figure 6:**
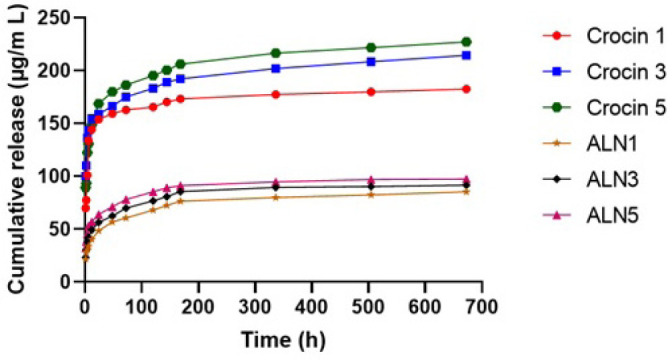
The release behavior of ALN and Crocin from the DBB scaffold (The concentration of the drugs is in grams per milliliter). ALN: Alendronate; DBB: Decellularized bovine bone

Release profile of ALN and crocin in DBB scaffolds *in vitro* is illustrated in Figure 6. A sustained release of ALN and crocin was observed for more than 168 hr. A burst release occurred within the first 12 hr for ALN and 24 hr for crocin. A sustained release of ALN and crocin can enhance bone healing and promote bone regeneration.

### Cytocompatibility evaluation

**Figure 7:**
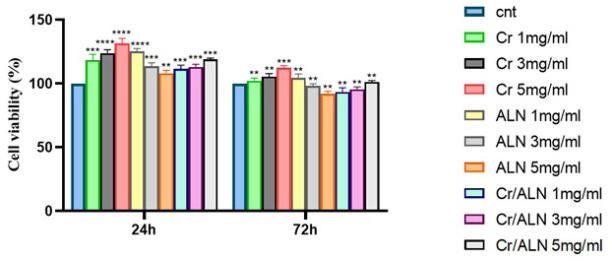
Investigating the cell viability of DBB scaffolds containing different amounts of ALN and crocin at two times, 24 and 72 hr

To determine the suitable concentrations of ALN and crocin for the DBB scaffold, an MTT assay was conducted. The results indicated that the viability of crocin at a concentration of 5 mg/ml was recorded at 131.23 ± 1.03% and 112.44 ± 2.01% after 24 and 72 hr, respectively. The viability of ALN at 1 mg/ml was measured at 123.71 ± 2.5% and 104.31 ± 1.04% after 24 and 72 hr. Additionally, the viability of the combination of crocin and ALN at 5 mg/ml was observed at 118.81 ± 3.14% and 101.53 ± 2.04% after 24 and 72 hr, respectively (Figure 7). 

### Blood compatibility evaluation

**Figure 8:**
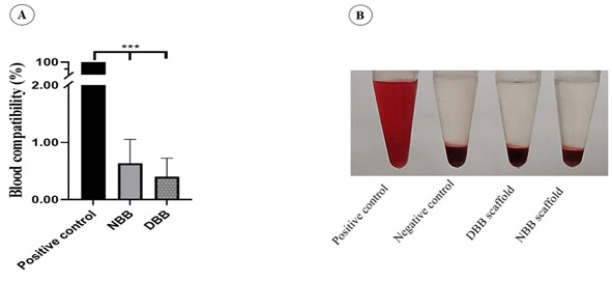
(A) The quantitative representation of hemolysis percentage in NBB and DBB compared to the positive control

The blood compatibility of NBB and DBB is illustrated in Figure 8. The results show that all scaffolds were compatible with blood, which is a noteworthy difference from the positive control group. The level of hemolysis of NBB and DBB scaffolds was 0.63 ± 0.21 and 0.4 ± 0.54%, respectively (*P*<0.001). The data was analyzed by one-way ANOVA.

### Osteogenesis assay

**Figure 9:**
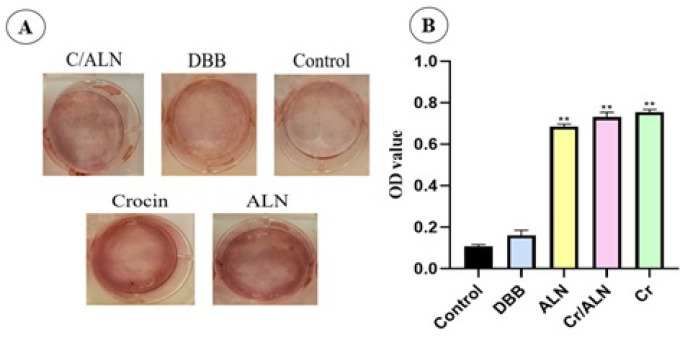
Alizarin red staining was conducted for the following groups: Cr, Cr/ALN, ALN, DBB, and control on day 7

The impact of conditioned media of the drug-loaded DBB scaffolds versus the control group on the differentiation of MC3T3E1 cells into osteoblasts was investigated in the absence of osteogenic media. ARS indicated more intense staining for Cr, Cr/ALN, and ALN groups on day 7 (Figure 9A). The absorbance values of Cr, ALN, and Cr/ALN were 0.755 ± 0.12, 0.732 ± 0.2, and 0.665 ± 0.5, respectively (Figure 9B).

### Expression of osteomarkers

**Figure 10:**
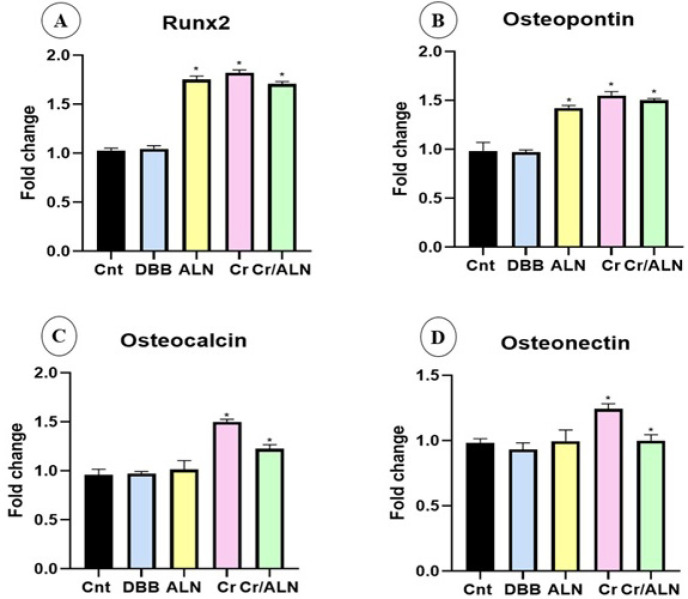
Expression of RUNX2 (A), osteopontin (B), osteocalcin (C), and osteonectin (D) in MC3T3-E1 cultured for 7 days

To assess the impact of drugs (Cr 5 mg/ml, Cr/ALN 5 mg/ml, ALN 1 mg/ml) on the differentiation of MC3T3 E1 cells based on MTT results, the expression quantities of selected osteogenic markers were evaluated after seven days (Figure 10). The expression of RUNX 2 showed a notable increase in groups Cr, Cr/ALN, and ALN, which were 70, 60, and 65%, respectively. The expression of Osteocalcin was 50% and 25% in groups Cr and Cr/ALN, respectively, while no changes were detected in the other groups. The expression level of Osteopontin increased by 50% in groups Cr, Cr/ALN, and 35% in the ALN group. Also, the expression of Osteonectin increased by 50% in the Cr group, while remaining unchanged in the other groups. Notably, there were no remarkable variations in the expression levels of all four genes between the control and DBB groups.

## Discussion

The remedy of severe bone defects, trauma, tumors, and congenital anomalies with critical size do not naturally heal spontaneously and require surgical interventions. Currently, this issue remains unresolved and is one of the most difficult challenges in orthopedic surgery. Therapeutic approaches for bone regeneration are limited to autograft, allograft, and metal implants. Although they have limitations, such as disease transmission, infection, immune system stimulation, toxic ion release, and the need for re-surgery in metal implants (32). Today, to overcome these limitations, bone tissue engineering proposes the preparation of decellularized bone grafts as a new approach for the repair of bone defects due to high mechanical resistance and stability, biocompatibility, the presence of growth factors, and the absence of immune system stimulation (33). The Ideal decellularization method is to eliminate Cell components while maintaining the structural integrity of the ECM. Previous studies have demonstrated that combining physical, chemical, and enzymatic methods can improve decellularization efficiency for hard tissues such as bone (34). For example, SDS has been shown to be highly effective for cell and DNA removal, although insufficient rinsing can leave residues that impair cell viability (35, 36). The delipidation step was performed using a chloroform–ethanol mixture, which is a widely recognized and cost-effective method for lipid extraction from bone tissue without compromising the structural integrity of the ECM or leaving cytotoxic residues, making it suitable for scaffold preparation in bone tissue engineering. (22). The use of physical methods for decellularization, such as ultrasonic treatment and freeze-thaw cycles, stands out for their ability to maintain mechanical properties (37). 

Reports in a study support that multi-step protocols combining SDS with enzymatic digestion and physical disruption can yield scaffolds free of visible cells and fat, with improved porosity compared to native bone (30, 38)

In our study, quantitative DNA analysis revealed a residual DNA content of 10.2 ± 2.3 ng/mg dry weight, which is well below the widely accepted threshold of 50 ng/mg for decellularized biomaterials. While this corresponds to ~20% of the DNA content of native bone, it falls within ranges reported for successfully decellularized xenografts in previous studies (39). Histological studies have confirmed that such approaches maintain collagen integrity while producing empty osteocyte lacunae, consistent with findings by Hensley *et al*. and Nam Minh Phuong Tran *et al*. (40, 41). Mechanical evaluations in other works, such as Tamilmahan *et al*., also indicate that careful integration of freeze–thaw cycles with chemical detergents can preserve scaffold strength better than chemical treatment alone (23).

Beyond structural integrity, the degradation rate of bone scaffolds is equally crucial for the stages of bone repair. Ideally, the degradation rate of bone scaffolds must be in line with the rate of bone regeneration at the defect site (42). 

Examination of drug release is essential to determine the quality and efficacy of decellularized scaffolds in the rate of drug release. The process of drug release is affected by various factors, such as the chemical composition of the scaffold, the rate of degradation, and the porosity of the scaffold (43, 44). Sustained release systems are particularly advantageous for bone healing, as they maintain bioactive concentrations over extended periods without the need for repeated administration (45). 

Biocompatibility and hemocompatibility of scaffolds were determined according to the ASTM F756 standard. Hemolysis rates of 0% to 2% are classified as non-hemolytic, rates between 2% and 5% are deemed slightly hemolytic, and rates exceeding 5% are categorized as hemolytic (46, 47) 

Additionally, the incorporation of bioactive molecules such as crocin and ALN has been explored in other systems for their osteoinductive and anti-resorptive effects, respectively. For instance, crocin has been associated with enhanced osteoblast differentiation and anti-oxidant activity, while ALN has been shown to modulate osteoclast function and promote bone matrix mineralization. In the present study, the concentrations of 1, 3, and 5 mg/ml for both Cr and ALN used in the MTT assay were selected based on previously reported ranges demonstrating dose-dependent effects on osteoblast viability and activity without inducing cytotoxicity(28, 48). For the osteogenesis assay, a 7-day culture period prior to Alizarin Red S staining was employed to evaluate early mineral deposition, as supported by studies reporting detectable calcium nodule formation within 5–7 days in osteogenically induced pre-osteoblastic cultures (49, 50). This timeframe was selected to capture early-stage mineralization while minimizing confounding effects from late-stage matrix degradation.

These bone-related markers include: RUNX 2, a transcription factor recognized as one of the earliest indicators of osteoblastic differentiation, which exhibits elevated expression primarily during the initial phases of differentiation (51). Osteonectin is a non-collagenous protein that is critically involved in the initiation and regulation of calcification (52). Osteocalcin, recognized as the most abundant non-collagenous protein found in bone, is produced during the initial phases of mineralization and serves as a marker for bone metabolic activity. Osteopontin plays a crucial role in the development and regulation of hydroxyapatite crystals (53). The observed upregulation of these markers indicates that DBB scaffolds containing the selected drugs effectively support bone formation.

**Table 1:** Primer sequences used for the amplification of osteogenic marker genes by RT-qPCR

β-actin	**forward 5’-ATATCGCTCCGCTCGTCGTC -3’** **reverse 5'- TACCAACCATCACACCCTGG -3’**
Osteocalcin	forward 5’- CAACCCCAATTGTGACGAGC -3’ reverse 5’- AACGGTGGTGCCATAGATGC -3’
Osteopontin	forward 5’- AGTGGTTTGCTTTTGCCTGT -3’ reverse 5’- GTGTTTGCTGTAATGCGCC -3’
Osteonectin	forward 5’- GATCAGCACCCGATTGATGG -3’ reverse 5’- AGGTCTCAAAGAAGCGAGTGG -3’
RUNX2	forward 5’- CGTCCCCATCCATCCATTCC -3’ reverse 5’- GAGGCAGAAGTCAGAGGTGG -3’

This table lists the forward and reverse primer sequences designed to amplify β-actin (housekeeping gene) and osteogenic differentiation markers including RUNX2, Osteocalcin, Osteopontin, and Osteonectin. These primers were used to evaluate gene expression in MC3T3-E1 cells cultured with Crocin, Alendronate, and Crocin/Alendronate-loaded decellularized bovine bone (DBB) scaffolds.

(A, B) SEM images show the surface morphology of native and (C, D) decellularized bone specimens. The images clearly show the pore structure on the surface of NBB and DBB. Notably, DBB exhibits considerably larger pore diameters and more interconnected pores than NBB. The scaffolds were examined using EDS surface analysis. This technique employed a representative spectrographic assessment to identify the surface chemical elements in (E) NBB and (F) DBB scaffolds

DBB: Decellularized bovine bone; EDS: Energy-dispersive X-ray spectroscopy; NBB: Native bovine bone

(A) Amount of residual DNA in the DBB scaffold compared to NBB. Total residual DNA was normalized by the dry weight of each bone specimen. Values are expressed as mean ± SD (n = 3 per group). Statistically significant differences are indicated as (**P<0.01). DAPI staining indicated (B) the presence of nuclei before decellularization and (C) after decellularization. DAPI: 4’,6-diamidino-2- phenylindole; NBB: Native bovine bone

(A) Native bovine bone, (B) decellularized bovine bone. (C x200, D x400) H and E staining showed NBB scaffolds, which displayed a high number of cells with visible nuclei (E x200, F x400) in contrast to the porous matrix in the DBB scaffolds with no cells or nuclei present. (G x200, H x400) Masson's trichrome staining indicated that lacunae containing osteocytes can be seen in NBB (I x200, J x400) scaffolds. The blue area on the DBB scaffolds implies that the collagen fibers and empty lacunae of osteocytes are visible. DBB: Decellularized bovine bone; NBB: Native bovine bone

Image representative of compressive engineering stress and engineering strain (SS) curves of NBB and DBB scaffolds (A). Force–displacement curve from the cyclic indentation curve of NBB and DBB scaffolds (B). DBB: Decellularized bovine bone; NBB: Native bovine bone

(A) The image shows that the DBB scaffold has higher porosity compared to the NBB scaffold. Values are represented as mean ± SD (n = 5; *P*<0.001). (B) percentage of weight loss for NBB, and DBB after (1, 2, 3, 7, 14, 21, 28, 35, 42, 49, 56, and 63 days). Bone scaffolds exhibited minimal weight loss after 63 days of incubation in PBS. ***P<0.001, ****P<0.0001

NBB: Native bovine bone; DBB: Decellularized bovine bone

(The concentration of the drugs is in grams per milliliter). In comparison to the control group. The results are presented as mean ± standard deviation. (n = 4; *P<0.05, **P<0.01, ***P<0.001, ****P<0.0001). ALN: Alendronate; DBB: Decellularized bovine bone

The data is presented as mean ± SD and *P*<0.001. ***P<0.001. Analyzed by the ANOVA test. (B) The clear supernatant observed in the NBB and DBB scaffolds confirms the compatibility of the scaffolds. NBB: Native bovine bone; DBB: Decellularized bovine bone

(A) OD of Alizarin red staining revealed no significant differences among the Cr, Cr/ALN, and ALN groups. In contrast, a remarkable difference was observed between the drug-loaded DBB scaffolds and both the DBB and control groups. The data are shown as mean ± SD, n=4, **P<0.01. ALN: Alendronate; DBB: Decellularized bovine bone

B-actin served as a reference to standardize the expression of each gene (n = 3; *P<0.05)

## Conclusion

In this study, the decellularization process was carried out with a combination of physicochemical and enzymatic methods to produce xenogenous scaffolds. The finding indicated that cell nuclei and lipids were effectively eliminated while maintaining the integrity of the ECM structure and collagen. Additionally, findings revealed that DBB scaffolds containing (Cr 5 mg/ml, Cr / ALN 5 mg/ml, ALN 1 mg/ml) have features such as osteogenic differentiation, degradability, mechanical stability, and non-toxicity. Consequently, these bioactive scaffolds represent a promising alternative intended for application in bone tissue engineering strategies.
